# Enhancement of the Refractory Matrix Diamond-Reinforced Cutting Tool Composite with Zirconia Nano-Additive

**DOI:** 10.3390/ma17122852

**Published:** 2024-06-11

**Authors:** Boranbay Ratov, Volodymyr A. Mechnik, Miroslaw Rucki, Edvin Hevorkian, Nikolai Bondarenko, Tetiana Prikhna, Viktor E. Moshchil, Vasyl Kolodnitskyi, Dmitrij Morozow, Aigul Gusmanova, Jerzy Jozwik, Makhiram Arshidinova, Arkadiusz Tofil

**Affiliations:** 1 Department of Geophysics, Institute of Geology and Oil and Gas K. Turysova, Satbayev University, Satpaev Str., 22, Almaty 050013, Kazakhstan; 2 V. Bakul Institute for Superhard Materials, National Academy of Science of Ukraine, Avtozavodska Str. 2, 04074 Kyiv, Ukraine; 3 Institute of Mechanical Science, Vilnius Gediminas Technical University, Sauletekio al. 11, LT-10223 Vilnius, Lithuania; 4 Faculty of Production Engineering, University of Life Sciences in Lublin, Gleboka 28, 20-612 Lublin, Poland; 5 Faculty of Mechanical Engineering, Casimir Pulaski Radom University, Stasieckiego Str. 54, 26-600 Radom, Poland; 6 Faculty of Engineering, Yessenov State University of Technology and Engineering, 32 mkr., Aktau 130000, Kazakhstan; 7 Department of Production Engineering, Mechanical Engineering Faculty, Lublin University of Technology, Nadbystrzycka 36, 20-618 Lublin, Poland; 8 Institute of Oil, Mining, Geology, and IT, Caspian University, Dostyk 85A, Almaty 050000, Kazakhstan; 9 Institute of Technical Sciences and Aviation, The University College of Applied Sciences in Chełm, ul. Pocztowa 54, 22-100 Chełm, Poland

**Keywords:** cutting tool, composite, particle reinforcement, microstructures, damage mechanics, sintering

## Abstract

This paper presents the results of the experimental research on diamond-reinforced composites with WC–Co matrices enhanced with a ZrO_2_ additive. The samples were prepared using a modified spark plasma sintering method with a directly applied alternating current. The structure and performance of the basic composite 94 wt.%WC–6 wt.%Co was compared with the ones with ZrO_2_ added in proportions up to 10 wt.%. It was demonstrated that an increase in zirconia content contributed to the intense refinement of the phase components. The composite 25 wt.%C_diamond_–70.5 wt.%WC–4.5 wt.%Co consisted of a hexagonal WC phase with lattice parameters *a* = 0.2906 nm and *c* = 0.2837 nm, a cubic phase (*a* = 1.1112 nm), hexagonal graphite phase (*a* = 0.2464 nm, *c* = 0.6711 nm), as well as diamond grits. After the addition of zirconia nanopowder, the sintered composite contained structural WC and Co_3_W_3_C phases, amorphous carbon, tetragonal phase *t-*ZrO_2_ (*a* = 0.36019 nm, *c* = 0.5174 nm), and diamond grits—these structural changes, after an addition of 6 wt.% ZrO_2_ contributed to an increase in the fracture toughness by more than 20%, up to *K*_Ic_ = 16.9 ± 0.76 MPa·m^0.5^, with a negligible decrease in the hardness. Moreover, the composite exhibited an alteration of the destruction mechanism after the addition of zirconia, as well as enhanced forces holding the diamond grits in the matrix.

## 1. Introduction

The WC–Co refractory matrix composites have been widely used in industrial applications, especially in mining and drilling tools, due to their high hardness, fracture strength, and wear resistance [1,2]. Diamond, being the hardest material with wear resistance and grinding ability, can serve as an ideal reinforcement for cemented carbide matrices to make up C_diamond_–WC–Co composites, which combine the high toughness of cemented carbide with the excellent wear resistance of diamond [3]. However, the properties of the composite depend on the proportion of its components, so researchers looked for an optimized content of carbon [4,5,6] and cobalt [7,8,9].

Cobalt is the most common binder for tungsten carbide composites [10], and its fraction and the carbide grain size determine the mechanical resistance and tribological behavior of the composite [11,12]. At the same time, the performance of the composites depends not only on the component proportion but also on the phase composition, microstructure, and morphology of the powders and grains. Realistic microstructures can be used for modeling the elastoplastic performance of various grades of WC–Co composites and to obtain good agreement between simulated and experimental data [13]. Garcia, with co-authors [14], conducted an extensive review of the effect of microstructures on the properties of cemented carbides, which, in turn, depend on the fabrication process [15,16]. Usually, tungsten carbide–cobalt composites are fabricated by liquid-phase sintering of powders mixed together [17]. For instance, the microwave-assisted sintering method allowed for improvement in the performance of cutting tools made out of WC–2Co and WC–4Co alloys [18,19]. There are also reports on additively manufactured WC–Co composites from the perspective of microstructural heterogeneity and mechanical properties [20]. In terms of composition, the addition of ultrafine WC powder to the WC–10Co composite increased its hardness, fracture toughness, and wear resistance [21]. Pittari, with co-authors, investigated WC–Co materials with similar cobalt binder contents but with variations in their microstructure, demonstrating respective differences in their properties [22]. Megret et al. [23] investigated the effect of the milling process on composite performance and found optimized parameters of high-energy ball milling of WC–10Co to obtain the desired porosity, grain size distribution, and mechanical properties after sintering. To modify the microstructure and, consequently, the mechanical properties of WC–Co-cemented carbides, some additives can be used [24]. Su et al. demonstrated an improvement in the mechanical characteristics and wear resistance of the WC–8Co composite after the addition of nano-alumina [25]. Yin et al. [26] investigated the effects of Cr_3_C_2_ and VC additions to the WC–6.0Co composite on WC’s grain size and shape and related mechanical properties. However, the main challenge for mining and rock-drilling tools remains, such as exposure to high temperatures under heavy loads and subsequent microstructural transformations in the matrix, which decrease its strength, wear resistance, and ability to hold diamond reinforcement.

In the present study, zirconia ZrO_2_ was chosen as an addition to the WC–Co matrix due to its high mechanical properties retained at elevated temperatures [27]. Moreover, it exhibits a transformation-toughening mechanism [28] when metastable at a room temperature tetragonal *t*-ZrO_2_ phase to the thermodynamically stable monoclinic *m*-ZrO_2_. In turn, the typical addition of Y_2_O_3_ stabilizer had an effect on the refinement of WC grains, improving the hardness and fracture toughness of the sintered composite [29]. Initial research demonstrated the feasibility of a modified spark plasma sintering method for WC–zirconia composites, with the optimal performance reached after electroconsolidation at 1350 °C under a mechanical pressure of 30 MPa for 2 min [30].

In mining applications, natural diamonds can be used as the cutter elements attached to steel blade structures, or in modern solutions, diamonds can be embedded in a tungsten carbide matrix body [31]. The cutting mechanism of these drill bits is a scrapping action, which is suitable for drilling rock formations. However, there are not many reports on the diamond-reinforced WC–Co–ZrO_2_ composites for this application. It was demonstrated previously that an addition of 4 wt.% and 10 wt.% of zirconia to the WC–Co composite with 25 wt.% of diamond powder reinforcement resulted in a smaller WC grain size and respective improvement in mechanical properties [32]. The grain refinement contributed to improved resistance to plastic deformation and tolerance to abrasion damage, as well as an enhancement of diamond grit retention being noted [33]. It was assumed that the addition of zirconia contributed to better adhesion between the diamond and matrix and to the appearance of squeezing residual stresses. In the frames of the study presented below, we analyzed the effect of a zirconia micropowder addition up to 10 wt.% on the phase formation during the sintering process and subsequent phase composition and microstructural features. These, in turn, have an effect on the hardness and fracture toughness of the composite 25C_diamond_–70.5WC–4.5Co, being highly beneficial to the structural transformation in the matrix under impact load. From the purpose-oriented perspective, the composite’s ability to keep retaining forces strong enough to hold the diamond grit in the matrix is among the most crucial properties.

## 2. Materials and Methods

### 2.1. Initial Powders

The refractory matrix composite samples were prepared using the tungsten carbide (WC), cobalt (Co), and zirconia (ZrO_2_) powders, as well as the diamond grit. WC powder of an average particle size of 2–8 μm and cobalt powder of a size of 2–3 μm were delivered by Kuybyshevburmash Industrial Enterprise (Samara, Russia). The cobalt powder was prepared according to the standard GOST 9721–79, ensuring a purity of Co of no less than 99.35 wt.%, with acceptable admixtures of Ni being no more than 0.4 wt.% and Fe below 0.2 wt.%. Partially yttria-stabilized zirconia (3 wt.% Y_2_O_3_) was delivered by the NANOE company (Ballainvilliers, France). The particle size of the zirconia powder exhibited a distribution between 50 nm and 1 μm, which qualified it as a nano-additive. The diamond powder delivered by the De Beers company (Johannesburg, South Africa) had a grit size of 500/400 and exhibited an average particle diameter of 0.45 mm.

### 2.2. Samples for Sintering

The research was divided into two main stages. One was devoted to the analysis of the refractory matrix properties dependent on the zirconia proportion, while the other stage was to check the interaction of the matrices with diamond reinforcement.

To assess the effect of the zirconia addition on the refractory matrix properties, the basic composite 94 wt.% WC–6 wt.%Co was prepared by mixing respective amounts of powders in alcohol and drying the blend in a thermal box. The sample sintered out of this blend was denoted #1, as shown in Table 1. In the case of the other samples (#2–#9) that contained zirconia additions, first, ZrO_2_ and Co powders were mixed together in alcohol until a homogenous blend was reached. Then, the respective amount of WC was added, carefully mixed, and dried. The compositions of the samples are shown in Table 1 in the order of increasing zirconia content.

The samples with diamond reinforcement (#10–#12) were prepared in the following way. The diamond-reinforced one without the zirconia addition, denoted #10, was obtained from the basic mixture (similar to sample #1) with the addition of 25 wt.% of diamond grit. It was important to keep the WC–Co proportion unchanged in order to correlate the properties of the matrix, investigated without diamond grit, and the ones of the diamond-reinforced composite. That is why, after the addition of 25% diamond filler, the overall percentage of the other components changed. The grit was moistened and added to the powder blend and then mixed together in an alcohol environment until homogeneity was reached. The diamond-reinforced samples with zirconia additions of 4 wt.% and 10 wt.% were prepared in a similar way, adding the diamond grit to the powder blends. The compositions of the samples can be seen in Table 1 above.

### 2.3. Sintering Method

The cylindrical composite samples of a diameter of 10 mm and a thickness of 5 mm were sintered using the modified spark plasma sintering method, patented [34] and described in detail in other papers [35]. The device applied an alternating current of 5000 A and voltage of 5 V directly to the powder mixture placed in the graphite mold under a vacuum of 6 Pa. The method allowed for obtaining heating rates as high as 400 °C/min, which, combined with a short holding time of 3 min in the sintering temperature of 1350 °C, ensured minimal grain growth. The powder blends underwent a mechanical pressure of 30 MPa, and the inner surfaces of the mold were lubricated with boron nitride to prevent an interaction between the sintered powder and graphite. A short sintering time and high heating rate were assumed to prevent diamonds from graphitization.

The as-obtained samples were ground so that the resulting cylinders had diameters of 9.62 mm and a height of 4.84 mm. Before the mechanical tests and structural analysis, the surfaces of the samples were polished until a mirror effect was reached. For that purpose, a diamond paste with a 1 μm particle size was used, as well as a colloidal solution with silicon oxide particles of a size of 0.04 μm.

### 2.4. Analysis of Sintered Specimens

To analyze the microstructure and elemental composition, as well as the fracture surfaces of the sintered samples, scanning electron microscopy (SEM) was used. The respective device, Mira 3 LMU, produced by the TESCAN company (Brno, Czech Republic), had a resolution of 1.2 nm. An energy dispersion microanalyzer, OXFORD X-MAX 80 mm^2^, was applied (Oxford Instruments, Tubney Woods, UK). The device ensured a measurement uncertainty of 0.01 wt.% for heavy metals and 0.10 wt.% for light metals. The accelerating voltage of 30 kV and Cu*K*α radiation with *λ*_Cu_ = 0.1542 nm were applied for the analyses of the sample surfaces.

To assess the phase composition, XRD analysis was performed using a Philips X’Pert Pro Materials Powder Diffractometer with a Cu*K*α source (Panalytical, Malvern, UK). The anode voltage was 45 kV, and the current was 40 mA. Diffractograms were made by coupled 2Θ-⍵ scans with a step of 0.025°. The data collection time at one point was 1 s. The phase concentration was determined through a full profile analysis with the Rietveld approach, using High Score Plus software [36]. The average level of deformations *ε* in the direction of the main optical axis *c* were determined from the angular positions and half-widths of the reflections (001)\(002) and (100)\(200) using the Williamson–Hall method [37,38]. The size of the coherent scattering region *D* was assessed with the same methodology.

In order to measure the Vickers hardness, a Falcon 500 microhardness tester (Innovates, Maastricht, The Netherlands) was used. It was equipped with a five-megapixel digital microscope that allowed for visualization of the indenter imprints after a load of 10 kg was applied and to measure the radial crack lengths necessary to determine the fracture toughness. To calculate the microhardness *H_V_* and resistance to crack propagation *K*_Ic_, the Falcon 500 microhardness tester was equipped with the Impressions software package, which performed an analysis of the measurement data in the semiautomatic mode.

To determine the dependence of the fracture toughness on the proportion of ZrO_2_ in the composite, it was necessary to apply similar loads of 10 kg in the microhardness test, which caused a crack appearance in every specimen. For each specimen, the fracture toughness *K*_Ic_ was calculated from 10 indentions. The microhardness *H_V_* was determined using the following formula:

(1) $$H_{V}=463.6\;\frac{F}{d_{mean}^{2}},$$

where *F* is the load on the indenter in N, and *d*_mean_ = (*d*_1_ + *d*_2_)/4 is half of the average length of the imprint diagonal in μm.

Fracture toughness *K*_Ic_ of the composite was determined according to the following expression [39]:

(2) $$\frac{K_{\mathrm{Ic}}\Phi }{H_{V}d^{0.5}}=0.15k{\left(\frac{C}{d}\right)}^{-1.5},$$

where Φ is the constraint factor (Φ ~ 3), *H_V_* is the Vickers hardness, *C* = (*C*_1_ + *C*_2_)/4 is the average length of the radial cracks measured from the center of the imprint, and *k* is a constant factor. The value of *k* = 3.2 was determined empirically from the *K*_I*c*_ values obtained for the macroscale samples by standard methods.

Considering the relationship for the Vickers hardness calculated from Equation (1) and the Equation (2) introduced by Evans and Charles [39], the final formula for the crack resistance appears as follows:

(3) $$KI_{c}=74.2\;\times \;10^{-2}\;\frac{F}{C^{1.5}}\;.$$

The deviations of the experimental values shown in the diagrams were calculated as square root deviations from the arithmetic mean, which can be considered the experimental standard deviation of the measurement.

## 3. Results and Discussion

### 3.1. Characterization of the Starting Materials

**Figure 1:**
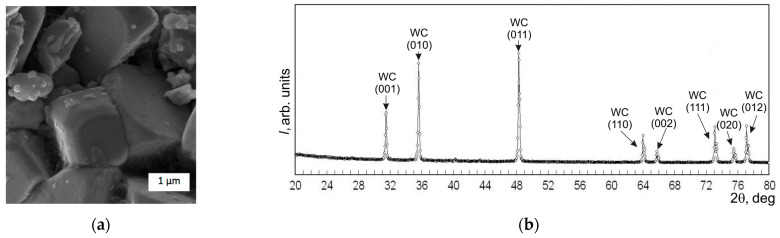
Starting powders of WC: (**a**) SEM image; (**b**) XRD diagram.

**Figure 2:**
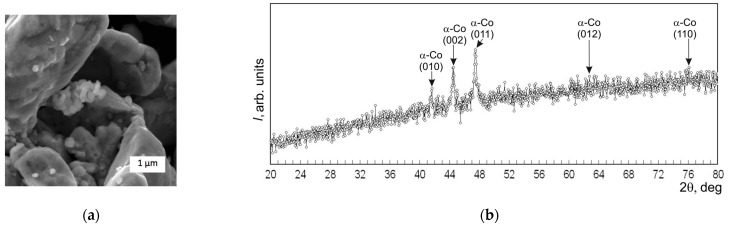
Starting powders of Co: (**a**) SEM image; (**b**) XRD diagram.

**Figure 3:**
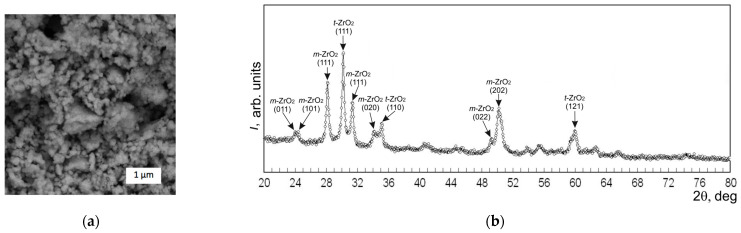
Starting powders of ZrO_2_: (**a**) SEM image; (**b**) XRD diagram.

Before making the sintered samples, the starting powders underwent XRD and SEM analyses. Figure 1, Figure 2 and Figure 3 show the SEM images and diffractograms of the tungsten carbide, cobalt, and zirconia, respectively. The magnification was kept the same in order to emphasize the difference between the particle sizes of the micropowders WC and Co compared to the ZrO_2_ nanopowder.

In the SEM image of the WC powder in Figure 1a, microcrystals can be seen in dimensions between 1 and 3 µm. Their form was mainly irregular, representing various cubes and prisms, and their structure was rather dense. In the overall mass, both smaller and larger particles were found, below 0.5 µm and above 3 µm. The XRD diagram in Figure 1b exhibits reflections corresponding most probably (99%) with WC of a hexagonal structure with the lattice parameters *a* = 0.29047 nm and *c* = 0.28355 nm. The rest of the 1% can be attributed to tungsten with a cubic structure and lattice parameter *a* = 0.31613 nm.

The starting powder of cobalt, seen in Figure 2a, consisted of irregular-shaped particles of dimensions between 1 and 3 µm. The majority of the particles exhibited a rounded form with smaller metal droplets on the surface. Smaller particles of cobalt formed larger agglomerates, and the high density of the bulk powder could be attributed to the rounded particles. Reflections in the XRD diagram corresponded to (100%) Co of a hexagonal structure with the lattice parameters *a* = 0.25097 nm and *c* = 0.40769 nm.

In turn, zirconia powder used as an additive exhibited agglomeration of two orders, seen in Figure 3a, composed out of small particles with dimensions difficult to assess. The first-order agglomerates are represented by the forms close to spheres of dimensions below 0.1 µm, which contributed to the high density of the bulk powder. The second-order agglomerates exhibited irregular polygonal and rounded forms of dimensions between 0.4 and 0.6 µm, with some of them reaching 1 µm. The agglomerates are built out of the smaller particles stuck together. When the powders were mixed together to obtain the blends listed in Table 1, the particles of each component remained generally the same, both in form and in dimensions.

The XRD diagram of the starting ZrO_2_ powder, shown in Figure 3b, exhibited lines corresponding with both tetragonal and monoclinic zirconia. The reflections corresponded mostly (60%) with *t*-ZrO_2_ of a hexagonal structure and with the lattice parameters *a* = 0.36105 nm and *c* = 0.51607 nm. The rest of the 40% corresponded with the monoclinic phase *m*-ZrO_2_ of lattice parameters *a* = 0.51657 nm, *b* = 0.53087 nm, and *c* = 0.52384 nm.

**Figure 4:**
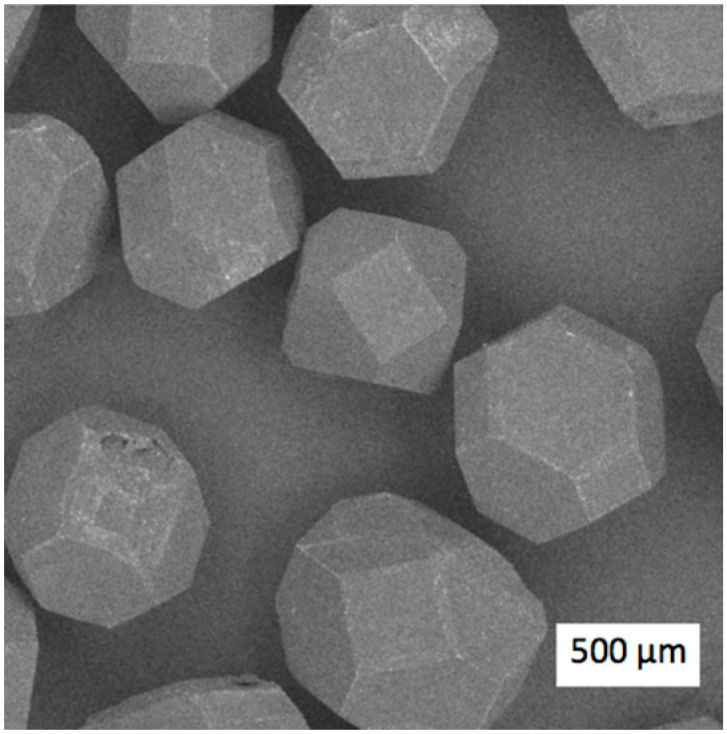
SEM image of the diamond particles used in the experiments.

The diamond grit had particle dimensions of 500 ± 360 μm, as is shown in Figure 4.

The diamond particles represented various simple, regular geometrical shapes with well-developed facets. The surfaces of the analyzed diamond particles had no distinguishable defects in the form of cracks or cavities.

### 3.2. Structural and Phase Composition after Sintering

**Figure 5:**
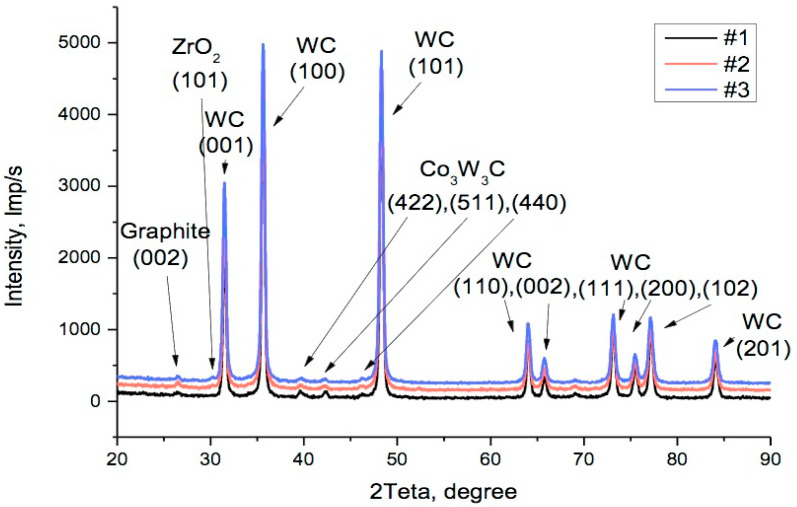
XRD diagrams of the sintered samples 94WC–6Co with zirconia additions: #1—no addition, #2—0.5 wt.%, and #3—1.0 wt.%.

**Figure 6:**
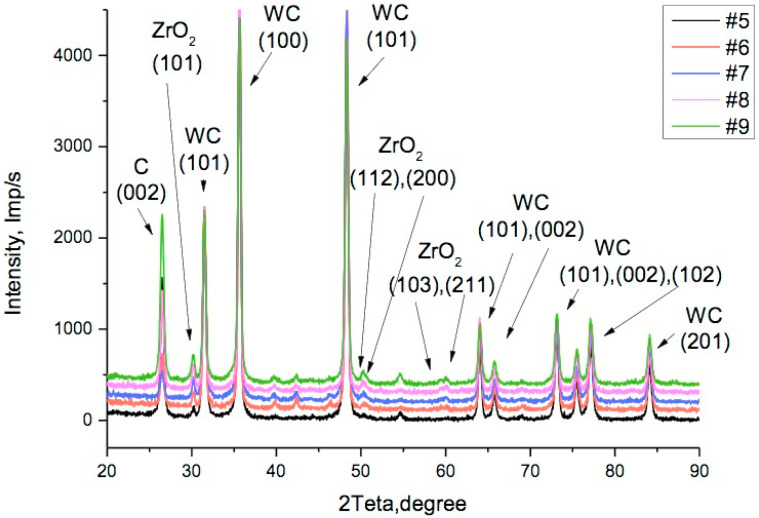
XRD diagrams of the sintered samples 94WC–6Co with zirconia additions: #5—2.0 wt.%, #6—4.0 wt.%, #7—6.0 wt.%, #8—8.0 wt.%, and #9—10.0 wt.%.

After the powder blends underwent sintering, they underwent XRD analysis. Figure 5 presents the diagrams for samples #1, #2, and #3, with respective contents of zirconia 0, 0.5 wt.%, and 1.0 wt.%. Diagrams for higher concentrations of zirconia in samples from #5 to #9 are shown in Figure 6. The dominant reflections belong to the *h-*WC phase No. 010-89-2727 with lattice parameters *a* = 0.2906 nm and *c* = 0.2837 nm, namely, reflections (001) at 31.5°, (100) at 35.6°, (101) at 48.3°, (110) at 64.0°, (002) at 65.7°, (111) at 73.1°, (200) at 75.4°, (102) at 77.1°, and (201) at 84.0°. Moreover, in Figure 5, the cubic phase of Co_3_W_3_C with lattice parameter *a* = 1.1112 nm can be seen under reflection No. 010-78-4940, (422) at 39.7°, (511) at 42.2°, and (440) at 46.1°. In position 2Ɵ = 26.5°, reflection (002) of the hexagonal graphite phase No. 030-65-6212 can be seen; its lattice parameters are *a* = 0.2464 nm and *c* = 0.6711 nm.

Starting from sample #2 with a small amount of zirconia added, a reflection (101) of the tetragonal zirconia phase No. 010-75-9645 with lattice parameters *a* = 0.36019 nm and *c* = 0.5174 nm appeared in the area 2Ɵ = 30.2°.

An increase in the ZrO_2_ proportion of up to 4 wt.% (sample #6) caused the appearance of doublets of weak reflections (112), (200) in the area 2Ɵ = 50°, as well as a doublet (103), (211) in the area 2Ɵ = 60°, as seen in Figure 6. These belong to a tetragonal *t-*ZrO_2_ phase with the symmetry group *P*4_2_/*mnc*. This group is transformable, i.e., under mechanical stress, it can undergo a martensitic transition to the monoclinic form, which in turn has an effect on the composite characteristics, in particular, on its fracture toughness and diamond grit retention ability. The *R_wp_* factor during analysis did not exceed *R_wp_* = 7.5. The results of the phase analysis and the phase mass share are shown in Table 2, while average values of coherent dispersion *D* and microdeformations *ε* in directions *c* and *a* were collected and displayed in Table 3.

The presence of the graphite phase may be explained partially by graphitization, but most of its amount can be attributed to the graphite from the mold. From the published data, it is known that the graphitization of the diamond is due to the high temperature accumulated on its surface, which is dependent on media, e.g., 850 °C in air, but in a vacuum, graphitization starts at 1200 °C [40]. There are reports demonstrating that the diamond exposed to heating for a long time may undergo graphitization in the temperature range of 650–750 °C [41]. Yan, with co-authors, investigated diamond behavior under instantaneous thermal shock, and they found that even at temperatures between 1500 °C and 1800 °C, the graphitization process took place mainly along the grain boundaries [42]. Thus, based on the available data, it is reasonable to assume that it was the sintering conditions with high heating rates that prevented the diamond grits from graphitization.

From Table 3, it can be concluded that an increase in the zirconia content caused the intensification of refinement of the phase components and average *ε* values for the composite 94WC–6Co. However, a maximal refinement corresponding with *D_c_* = 18.2 nm and *D_a_* = 24.0 nm and respective microdeformations in directions *c* and *a* took place in sample #7, where zirconia occupied 6 wt.%. A further increase in the Co content weakened the fracture toughness due to the differences in the thermal expansion coefficient. Fracture toughness is discussed in Section 3.3, demonstrating that the effect of a zirconia addition stays in agreement with the data collected in Table 3.

Thus, XRD analysis with the Williamson–Hall method provided a reliable explanation for enhanced diamond grit retention by the 94WC–6Co matrix after the addition of zirconia due to the appearance of the compressive microstresses in the structure.

### 3.3. Microhardness and Fracture Toughness

**Figure 7:**
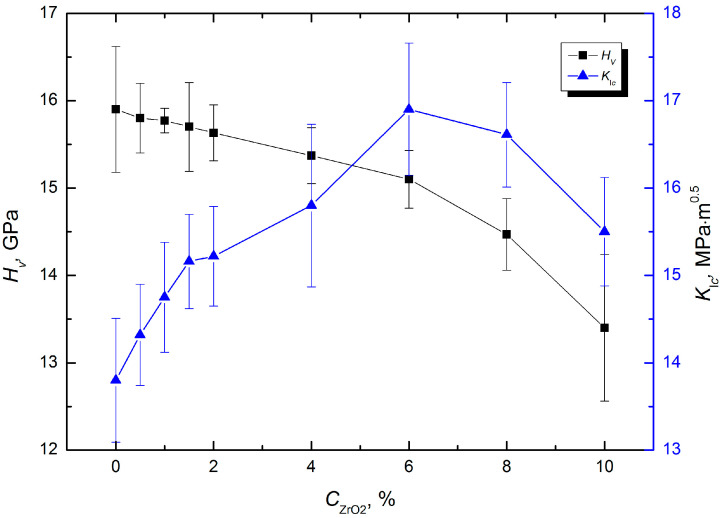
Dependence of microhardness *H_V_* and fracture toughness *K*_Ic_ on zirconia additions content *C*_ZrO2_.

Obviously, the observed alterations in the phase composition of the sintered WC–Co composite specimens with different proportions of zirconia additive should have a certain effect on the mechanical properties. The most significant changes could be expected in the case of specimens with a 6 wt.% of ZrO_2_, which corresponds with sample number #7 (Table 1). And indeed, some decrease in microhardness was observed for higher proportions of zirconia, which is seen in Figure 7.

The curve representing *H_V_* exhibited three areas of different declination angles. Namely, for the zirconia content from 0 up to 6 wt.%, the microhardness decreased by 0.8 GPa, from 15.9 ± 0.72 GPa down to 15.1 ± 0.33 GPa, which gives the ratio of microhardness to zirconia concentration changes Δ*H_V_/*Δ*C*_ZrO2_ = 0.13 [GPa/wt.%]. A further increase in zirconia up to 8 wt.% caused a decrease in hardness, but now from 15.1 ± 0.33 GPa down to 14.7 ± 0.41 GPa, with a ratio of Δ*H_V_/*Δ*C*_ZrO2_ = 0.2 [GPa/wt.%]. Finally, an increase in zirconia content from 8 wt.% up to 10 wt.% decreased the hardness from 14.7 ± 0.41 GPa down to 13.4 ± 0.84 GPa, with a ratio of Δ*H_V_/*Δ*C*_ZrO2_ = 0.65 [GPa/wt.%].

The abovementioned dependence can be attributed to the dispersion of the initial powders, which leads to the formation of agglomerates during the mixing process. In addition, a zonal separation phenomenon during sintering and the formation of micropores could have contributed to the described effect. The presence of porosity usually leads to an additional decrease in the hardness of the material, which can negatively affect the performance of composites.

In contrast, the addition of zirconia to the WC–Co composite in proportions from 0 up to 6 wt.% had a positive effect on the fracture toughness. Compared to a reduction in microhardness *H_V_* by 5%, the *K*_Ic_ of the composite increased by 21%, as shown in the blue curve in the diagram in Figure 7. However, a further increase in the zirconia content *C*_ZrO2_ caused a worsening of the fracture toughness.

It is worth noting that the specimens with a proportion of *C*_ZrO2_ = 6 wt.% that reached a maximal fracture toughness of *K*_Ic_ = 16.9 ± 0.76 MPa·m^0.5^ constituted a critical point between the areas with the ratios Δ*H_V_/*Δ*C*_ZrO2_ = 0.13 [GPa/wt.%] and 0.2 [GPa/wt.%]. The importance of this observation is connected to the fact that usually hardness and fracture toughness exhibit opposite responses to the structural changes in the material.

Thus, the WC–Co composite specimens with a proportion of *C*_ZrO2_ = 6 wt.% attracted the most interest due to their high microhardness and fracture toughness. Its characteristics can be explained from the perspective of the Hall–Petch law considering the grain size to be the main factor determining the fracture toughness of a composite. In fact, when *C*_ZrO2_ was increased from 0 up to 6 wt.%, the average grain size decreased by ca. 50%.

The observed effect also correlated with the phase analysis of the sintered specimens. The data collected in Table 3 demonstrated that an increase in the zirconia content caused more intense refinement of the phase components and average lattice microdeformations ε in directions *c* and *a*. Notably, the maximal refinement of the phase components with *Dc* = 18.2 nm and *Da* = 24.0 nm, as well as minimal values of the microdeformations *ε* in directions *c* and *a* of the 94WC–6Co composite took place when the content of ZrO_2_ in it was 6 wt.%.

The findings remain in agreement with other reports concerning the effect of CrB_2_ additives on the WC–Co composites’ performance. For instance, an increase in CrB_2_ proportion up to 4 wt.% caused an improvement in *K*_I_*_c_* from 9.8 up to 14.5 MPa·m^0.5^ accompanied by a slight decrease in the microhardness from 15.1 GPa down to 13.9 GPa [43]. Noteworthy, typical values of *K*_I_*_c_* and *H_V_* in the composites 94 wt.%WC–6 wt.%Co, obtainable using various sintering techniques, reach 12.0–14.3 MPa·m^0.5^ and 14.0–15.0 GPa, respectively [44,45]. The tested composite 88.36 wt.%WC–5.64 wt.%Co–6.0 wt.%ZrO_2_ (specimen #7) exhibited significant improvements in these parameters, which can be attributed to the complex combination of transformation and dispersion strengthening mechanisms, as well as structural and phase composition peculiarities. Presumably, it would have an effect on the performance of the composite, in particular, retention of the diamond grits in the refractory matrix.

### 3.4. Elemental Composition and Porosity Analysis

It is important to note that the investigated samples did not exhibit porosity, especially for the contact area between the diamond and the matrix, which contains no micropores, microcracks, or discontinuities. It can be attributed to the properties of zirconia crystals, especially their high fracture toughness and mechanical strength at elevated temperatures [27]. In addition, ZrO_2_ exhibited a transformational hardening mechanism [28] due to phase transformations between the metastable tetragonal *t*-ZrO_2_ and thermodynamically stable monoclinic *m*-ZrO_2_. This transition is accompanied by changes in the specific volume of the phases and the appearance of the squeezing mechanical stresses that prevent crack propagation.

**Figure 8:**
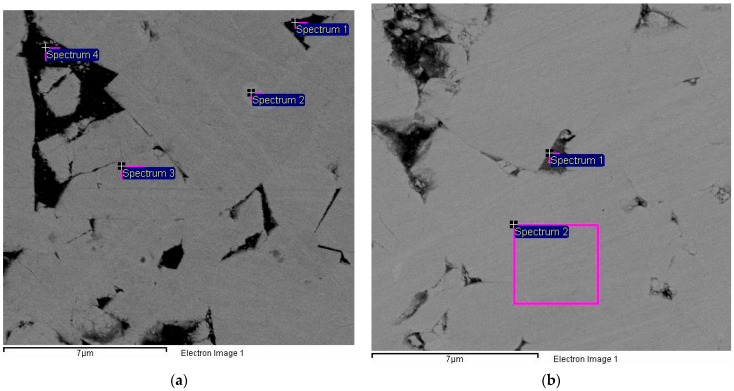
SEM images of sample surfaces with areas chosen for elemental analysis: (**a**) sample #1, 94WC–6Co; (**b**) sample #9, 86.6WC–5.4Co–10.0ZrO_2_.

Comparative analyses of the interfacial areas revealed a principal difference between the damaged structures of the composites sintered with and without ZrO_2_ addition. Table 4 and Table 5 contain the results of the elemental analysis conducted for the spectra marked in Figure 8. Figure 8a and Table 4 represent sample #1 without the zirconia addition, while Figure 8b and Table 5 illustrate sample #9 of composition 86.60WC–5.4Co–10.0ZrO_2_.

### 3.5. Fracture Analysis of the 94WC–6Co Diamond-Reinforced Composites

**Figure 9:**
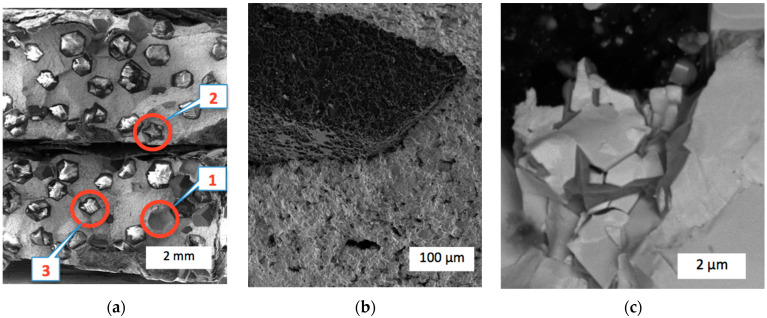
SEM images of the fracture surface of 25C_diamond_–70.5WC–4.5Co composite: (**a**) Overall view in edge contrast with holes after diamond grits torn out (1), undamaged grits (2), and damaged ones (3); (**b**) a hole after diamond grit torn out (edge contrast); (**c**) a damaged diamond grit (compositional contrast).

The results of the XRD analysis corresponded with the observations of the fracture surface structures of the broken samples C_diamond_–(WC–Co)–ZrO_2_. Figure 9 shows examples of the SEM images of the fracture surface of sample #10 composed out of 25C_diamond_–70.5WC–4.5Co without zirconia additives. For a better illustration, the images are given in different scales and either in compositional or edge contrasts, emphasizing important details of the surfaces. Notably, the fracture took place along the diamond–matrix interfaces, which is confirmed by the holes after the diamond grits were torn out and the undamaged diamonds remained in the matrix material, as is seen in Figure 9a,b. However, several diamond grits exhibited damage, as presented in Figure 9c.

Obviously, interfacial damage weakened the composite, preventing full exploitation of the hard diamond reinforcement in the refractory matrix. Some diamond grits appeared to be damaged, showing high retention forces in the matrix. However, in any case, the refractory matrix exhibited features typical for brittle damage with smooth fracture surfaces. The grain size of the matrix may be assessed in a range from 3 μm up to 8 μm.

**Figure 10:**
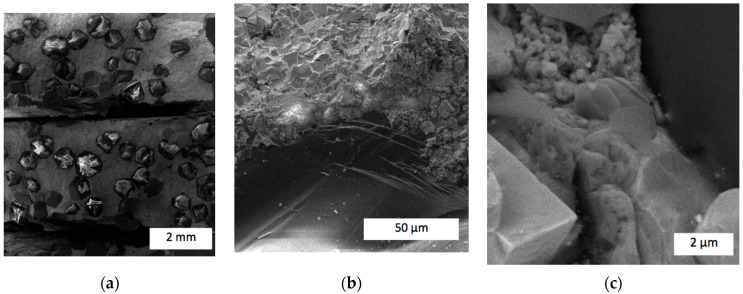
SEM images of the fracture surface of 25C_diamond_–66.74WC–4.26Co–4ZrO_2_ composite in edge contrast: (**a**) Overall view; (**b**) an interface between the matrix and diamond grit; (**c**) a damaged matrix surface.

Analysis of the fracture surface of the composite with the zirconia addition 25C_diamond_–66.74WC–4.26Co–4ZrO_2_ (sample #11), shown in Figure 10, revealed much fewer holes than sample #10 with no ZrO_2_. That proved a smaller role of the interfacial damage and better retention of the diamond grits in the matrix.

**Figure 11:**
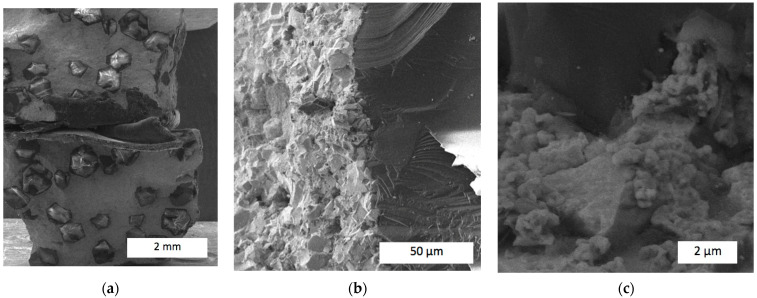
SEM images of the fracture surface of 25C_diamond_–61.1WC–3.9Co–10ZrO_2_ composite in edge contrast: (**a**) Overall view; (**b**) an interface between the matrix and diamond grit; (**c**) a damaged matrix surface.

Thus, the addition of 4 wt.% of zirconia enhanced the interface between the diamond grits and the refractory matrix, strengthening the retention of the diamond reinforcement. From the perspective of the harsh work conditions that rock-cutting tools are used in, strong bonds between the diamond reinforcement and refractory matrix have an effect on the wear mechanism. When the diamond grit is torn out of the matrix, it increases the scratching forces, destroying the matrix [46]. The images presented in Figure 10b,c also indicate different damage mechanisms of the matrix itself. First of all, nanopores of dimensions between 100 and 200 nm were found. Moreover, apart from the smooth surfaces damaged by brittle cracks, the fracture had areas with irregular cavities caused by viscous damage mechanisms. These features certainly contributed to the improved wear resistance of the diamond-reinforced composite. A further increase in zirconia content improved both the diamond retention and damage features. Figure 11 presents SEM images of the fracture surface of sample #12 with 10 wt.% of zirconia. No holes were present after torn-out diamond grits were found throughout the fracture surface, proving that no interfacial damage between the matrix and reinforcement took place. Compared to the samples presented in Figure 9 and Figure 10, this demonstrated the highest retention force generated by the addition of 10 wt.% ZrO_2_.

Moreover, the addition of 10 wt.% ZrO_2_ further altered the damage mechanism, compared to the 4 wt.% proportion. The structure of the refractory matrix fracture surface of sample #12 exhibited features typical of viscous damage, with developed pits and hills throughout the fracture surface. Microstructural features of the fracture surface are also seen on the surfaces of damaged diamond grits. In composite sample #10 without the zirconia addition, damaged diamond grits usually indicated a single source of the breaking stress placed in the contact area with the matrix. In turn, the composite 25C_diamond_–61.1WC–3.9Co–10ZrO_2_ exhibited numerous excessive stresses within a diamond grit body being squeezed by the refractory matrix. The surface of the broken diamond, seen in Figure 11b, exhibited developed cracks that form a microscale relief with a highly dense network of crack branches.

It can be concluded that the diamond-reinforced composite, with no zirconia addition, dominated the formation of either large cobalt matrix areas or direct contact zones with grains of tungsten carbide. In turn, when zirconia was added, long and thin and approximate 100 nm layers of cobalt phase can be found, even between the small WC grains. This structural feature contributed to the increased plasticity and improved performance of the composite.

Moreover, the observed microstructural features of the composite contributed to better retention of the diamond grits in the refractory matrix. In the experiments, the composition 25C_diamond_–61.1WC–3.9Co–10.0ZrO_2_ exhibited the best diamond-retaining ability, which is very important from the perspective of rock-drilling tool applications. Based on the performed experimental research, it can be stated that an increase in zirconia content, at least up to 10 wt.%, contributed to an increase in the plasticity and density of the matrix structure, which in turn led to the enhancement of the diamond retention forces.

Suppression of the grain growth in the structure of polycrystalline diamond materials is an important issue [29]. It was demonstrated previously [32] that the addition of 4 wt.% and 10 wt.% of zirconia to the composite 25C_diamond_–70.5WC–4.5Co prevented grains from so-called Ostwald ripening and acts as a grain growth inhibitor, which resulted with smaller WC grain sizes. The grain refinement contributed to the improved performance, which was measured using modulus *E* and nanohardness *H* values to calculate the elastic strain-to-failure ratio *H*/*E*, resistance to plastic deformation index *H*^3^/*E*^2^, and index of tolerance to abrasion damage 1/(*E*^2^*H*) [33]. All these indexes showed continuous increases for higher zirconia percentages, from 0 up to 10 wt.%, which is consistent with the presented results of this study.

Perhaps the most important achievement is the improvement in diamond retention forces. Most of the published papers do not address this important issue since it is a challenging task to ensure good adhesion between the diamond surface and the matrix. In some papers, like in [47], SEM images of the fracture surface clearly exhibited slots, discontinuities, pores, and other defects that obviously weakened diamond retention. In our research, Figure 10 and Figure 11 proved that in composites 25C_diamond_–66.74WC–4.26Co–4ZrO_2_ and 25C_diamond_–61.1WC–3.9Co–10ZrO_2_, holding forces between the matrix and diamond grit may be stronger than that in the diamond grit itself.

Since high quality, structural reliability, and durability are the most important requirements for production [48], more investigations are planned in this direction. In further research, the wear resistance in work conditions will be investigated. Because of the presence of diamond grit in the composite, especially with so high retaining forces, laboratory experiments cannot provide reliable data on wear. The counter-bodies usually used for wear assessment quickly become worn out, leaving the reinforcement in the 25C_diamond_–70.5WC–4.5Co composite almost intact. Thus, it is planned to fabricate a series of drill bits with the investigated composite and to measure its working time and wear when drilling through the reference rock material in a laboratory or through certain rocks in field conditions.

## 4. Conclusions

The experimental study on the zirconia addition to the composite 25C_diamond_–70.5WC–4.5Co demonstrated its effect on phase formation, structural modifications, fracture mechanisms, and diamond grit retention forces. In particular, it can be stated as follows:

The sintered basic composite 94WC–6Co consisted of a hexagonal WC phase, a cubic Co_3_W_3_C phase, as well as a hexagonal graphite phase.

1. The basic composites with the addition of ZrO_2_ from 0.5 wt.% up to 10.0 wt.% consisted of structural phases WC, Co_3_W_3_C, amorphous carbon, and a tetragonal zirconia phase. In the composites with a zirconia percentage above 1 wt.%, an intense refinement of the phase components was observed. Microdeformations *ε* in directions *c* and *a* of the composite 94WC–6Co appeared to be decreased, which could be attributed to the specific phase composition.
2. The Williamson–Hall method allowed us to determine the maximal refinement corresponding with *D_c_* = 18.2 nm and *D_a_* = 24.0 nm, as well as respective microdeformations *ε_c_* = 0.003% and *ε_a_* = 0.013% in directions *c* and *a*. It took place in the composite, where zirconia occupied 6 wt.%.
3. Increased diamond retention forces in composites with zirconia additions could be attributed to the presence of a large amount of tetragonal ZrO_2_ phase. This phase ensured the transformational mechanism of the matrix enhancement, densification of its structure, as well as refinement of the refractory matrix structure. In particular, the formation of thin (ca. 100 nm) cobalt interlayers between WC grains largely contributed to the enhancement of the composite.

The presented initial results make it possible to start in situ experiments with cutting tools to assess their durability and performance in harsh conditions of rock drilling.

## Figures and Tables

**Table 1:** Compositions of the powder samples prepared for sintering, wt.%.

№	C_diamond_	WC	Co	ZrO_2_
#1	-	94	6	-
#10	25	70.5	4.5	-
#2	-	93.53	5.97	0.5
#3	-	93.06	5.96	1.0
#4	-	92.59	5.91	1.5
#5	-	92.12	5.88	2.0
#6	-	90.24	5.76	4.0
#11	25	66.74	4.26	4.0
#7	-	88.36	5.64	6.0
#8	-	86.48	5.52	8.0
#9	-	84.60	5.4	10.0
#12	25	61.1	3.9	10.0

**Table 2:** Phase compositions of the sintered samples.

Sample №	WC, wt.%	Co_3_W_3_C, wt.%	Graphite, wt.%	ZrO_2_, wt.%
#1	97.97	1.26	0.77	0.00
#2	97.41	1.36	0.72	0.51
#3	96.83	1.55	0.68	0.94
#5	94.69	0.00	4.16	1.15
#6	94.68	1.39	1.60	2.33
#7	94.16	1.46	0.89	3.49
#8	91.37	1.35	3.27	4.01
#9	91.39	1.35	3.25	4.01

**Table 3:** The values of coherent dispersion *D* and microdeformations *ε* in directions *c* and *a*.

Sample №	*D_c_*, nm	*ε_c_*, %	*D_a_*, nm	*ε_a_*, %
#1	26.5	0.070	29.3	0.057
#2	26.4	0.145	25.4	0.065
#3	20.8	0.020	23.4	0.031
#5	20.6	0.067	25.3	0.035
#6	24.2	0.029	27.8	0.024
#7	18.2	0.003	24.0	0.013
#8	23.1	0.032	27.1	0.025
#9	22.7	0.084	23.4	0.029

**Table 4:** Elemental compositions of the point spectra and total for the composite 94WC–6Co.

Spectrum *	Elemental Composition, %					
	Instats.	C	O	Co	W	Total
Spectrum 1	Yes	10.43	0.97	79.09	9.51	100.00
Spectrum 2	Yes	10.96	0.81	-	88.23	100.00
Spectrum 3	Yes	11.61	-	-	88.39	100.00
Spectrum 4	Yes	13.94	2.67	65.14	18.15	100.00
Max.		13.94	2.67	79.09	88.39	
Min.		10.43	0.81	65.14	9.51	

* Spectra correspond with areas marked correspondingly in Figure 8a.

**Table 5:** Elemental compositions of the point spectra and total for 86.6WC–5.4Co–10.0ZrO_2_.

Spectrum *	Elemental Composition, %					
	Instats.	C	O	Co	W	Total
Spectrum 1	Yes	9.51	2.39	49.57	38.53	100.00
Spectrum 2	Yes	9.21	-	-	90.79	100.00
Max.		9.51	2.39	49.57	90.79	
Min.		9.21	2.39	49.57	38.53	

* Spectra correspond with areas marked correspondingly in Figure 8b.

## Data Availability

Data are contained within the article.
